# Fatal Injuries in Offshore Oil and Gas Operations — United States, 2003–2010

**Published:** 2013-04-26

**Authors:** Matthew M. Gunter, Ryan Hill, Mary B. O’Connor, Kyla D. Retzer, Jennifer M. Lincoln

**Affiliations:** Office of Safety, Health, and Working Conditions, Bureau of Labor Statistics, US Dept of Labor; Western States Office; Alaska Pacific Office, National Institute for Occupational Safety and Health, CDC

During 2003–2010, the U.S. oil and gas extraction industry (onshore and offshore, combined) had a collective fatality rate seven times higher than for all U.S. workers (27.1 versus 3.8 deaths per 100,000 workers). The 11 lives lost in the 2010 Deepwater Horizon explosion provide a reminder of the hazards involved in offshore drilling. To identify risk factors to offshore oil and gas extraction workers, CDC analyzed data from the Bureau of Labor Statistics (BLS) Census of Fatal Occupational Injuries (CFOI), a comprehensive database of fatal work injuries, for the period 2003–2010. This report describes the results of that analysis, which found that 128 fatalities in activities related to offshore oil and gas operations occurred during this period. Transportation events were the leading cause (65 [51%]); the majority of these involved aircraft (49 [75%]). Nearly one fourth (31 [24%]) of the fatalities occurred among workers whose occupations were classified as “transportation and material moving.” To reduce fatalities in offshore oil and gas operations, employers should ensure that the most stringent applicable transportation safety guidelines are followed.

CFOI, a cooperative program between the BLS and state governments, is the most comprehensive national surveillance system for work-related fatalities in the United States. Multiple data sources are used to collect information on each fatality. A fatal injury is considered work-related if the event leading to the injury occurred while the employee was working, based on confirmation by two independent sources.

The oil and gas extraction industry includes three types of companies, defined according to the North American Industry Classification System (NAICS): oil and gas operators who control and manage leased areas (NAICS 211), drilling contractors who drill the wells (NAICS 213111), and well-servicing companies who provide all other types of support operations that prepare a well for production and completion (NAICS 213112). Offshore oil and gas operations include all activities involved in the extraction of crude oil and natural gas from reservoirs found beneath the seafloor. CFOI does not include a variable to specifically identify offshore fatalities. Further, not all workers involved in offshore operations are directly employed in the oil and gas extraction industry, and therefore would not be captured in one of the three NAICS codes above.

To accurately identify all workers killed during offshore oil and gas operations, CDC and BLS identified cases two ways: 1) the fatality was included in one of the industry’s three NAICS codes, and the CFOI variable denoting the location was coded as a body of water, or 2) the fatality contained any one of the following key words in the CFOI narrative: “offshore,” “off shore,” “platform,” “boat,” “ship,” “barge,” or “helicopter,” and further examination of the case revealed that the incident was related to offshore oil and gas operations. Cases identified during 2003–2010 were analyzed by year, age, race/ethnicity, event type, nature and source of injury, and NAICS code. CFOI narrative data were reviewed to identify factors involved in helicopter events. Annual fatality rates were calculated using a count of active offshore drilling rigs as the denominator, which included fixed and semisubmersible drilling rigs, mobile offshore drilling units, and drillships. A Poisson regression model was used to measure trends.

During 2003–2010, a total of 128 fatalities occurred in activities related to offshore oil and gas operations in the United States, an average of 16 per year. All but one fatality occurred in Gulf of Mexico operations. All decedents were male with a mean age of 41.4 years. The majority were non-Hispanic whites (101 [79%]). Despite a 63% decrease in the number of active offshore drilling rigs during 2003–2010, the number of annual fatalities during offshore operations remained stable, resulting in a statistically significant increase in the number of fatalities per rig rate (Figure).

Transportation events were the leading cause of fatalities (65 [51%]), followed by contact with objects or equipment (21 [16%]), fires and explosions (17 [13%]), and exposure to harmful substances/environments (16 [13%]) (Table). Seventy-five percent of transportation events were associated with aircraft, all of which were helicopters (49 fatalities). Seventeen helicopter events occurred; 11 of these resulted in 43 (88%) of the fatalities. CFOI narratives noted that mechanical failure or loss of engine power was associated with five events (eight fatalities), and bad weather played a role in three of the events (seven fatalities). In five events, a total of nine fatalities involved occupants who survived the initial impact but later drowned. All of the helicopter events occurred in Gulf of Mexico offshore operations.

Two thirds of the fatalities involved workers employed in the oil and gas extraction industry (87 [68%]). Of those, half involved workers employed by well servicing companies (43 [49%]), followed by drilling contractors (26 [30%]), and oil and gas operators (18 [21%]). The remainder involved workers in offshore oil and gas operations who were classified as employees in another industry, including transportation and warehousing (23 [18%]), construction (10 [8%]), and all other industries (eight [6%]). Nearly one fourth (31 [24%]) of the decedents worked in occupations classified as “transportation and material moving” that transported workers and their equipment to and from offshore drilling platforms.

## Editorial Note

Catastrophic events like the Deepwater Horizon explosion attract intense media attention but do not account for the majority of work-related fatalities during offshore operations. This report found that transportation events (specifically helicopter crashes) were the most frequent fatal event in this industry.

The findings in this report are consistent with previously reported data. Mechanical failures and bad weather were identified as the most common factors in helicopter crashes related to offshore oil and gas operations in the Gulf of Mexico during 1983–2009 (1). That study also found that two thirds of all forced or precautionary landings resulting from mechanical failures occurred in water; aircraft floatation devices either failed to deploy or malfunctioned in 20% of nonfatal crashes (1). Another study analyzed Canadian civilian helicopter crashes into water and found that lack of warning time, sinking, and helicopter inversion were major contributing factors in fatalities (2). The same study found that drowning was the primary cause of death in helicopter crashes over water and, even when available, use of life jackets among pilots and passengers was inconsistent (2). Other studies also indicate drowning and exposure as post-impact hazards to survival (3,4).

To increase pilots’ situational awareness and improve safety, the Federal Aviation Administration (FAA) worked with the oil and gas industry and aircraft operators in the Gulf of Mexico to implement Automatic Dependent Surveillance-Broadcast (ADS-B) technology, which uses satellites to transmit information to air traffic controllers and to other aircraft equipped with ADS-B avionics (5). This technology provides flight tracking, improved communications capabilities, enhanced weather information, and terrain and traffic information. Before the implementation of this technology, radar coverage did not pick up low-flying aircraft and traditional radio communications had limited capability, and therefore were not effective in warning pilots of rapidly changing weather conditions. Since late 2009, when ADS-B was implemented in the Gulf of Mexico, no weather-related fatal helicopter crashes during oil and gas operations have occurred as of the end of 2012 (6).

What is already known on this topic?

The oil and gas extraction industry has an elevated occupational fatality rate that is consistently among the highest of any U.S. industry. The causes of the most frequent fatalities among onshore oil and gas extraction workers are well known. However, little is known about the unique risk factors faced by workers during offshore oil and gas operations.

What is added by this report?

During 2003–2010, a total of 128 fatalities occurred among offshore oil and gas workers. Transportation fatalities (65 [51%]) were the most common. A total of 49 (75%) transportation fatalities were associated with helicopters. All of the helicopter fatalities occurred in Gulf of Mexico operations.

What are the implications for public health practice?

Employers should ensure that the transportation safety guidelines developed by the International Association of Oil and Gas Producers are followed. Pilots and passengers should wear life jackets during flights over water and complete helicopter underwater escape training, and helicopters should be equipped with survival equipment specific to their operating environment.

The findings in this report are subject to at least two limitations. First, the level of detail and quality of narrative source information in CFOI used to identify one third of the fatalities in this report and identify factors related to helicopter events might vary from case-to-case. A narrative might exclude important information if it was not included in the source documents used to develop the CFOI case record. Conversely, mention of a given factor in the CFOI case record does not necessarily mean that the factor caused or contributed to the incident. Second, occupational fatality rates based on the number of offshore workers or the number of offshore flight hours could not be calculated because those data were not available. As a result, the number of active offshore drilling rigs, an estimate of industry activity that does not include offshore producing platforms, was used to calculate occupational fatality rates.

To reduce fatalities in the offshore oil and gas industry, employers should ensure that the most stringent applicable transportation safety guidelines are followed. The International Association of Oil and Gas Producers (OGP) has developed guidelines for aircraft operations in the oil and gas industry that exceed FAA safety regulations (7). According to the OGP guidelines, pilots and passengers should complete helicopter underwater escape training and wear life jackets during flights over water. Floatation gear fitted to the helicopter should automatically inflate on impact with water and be capable of supporting the helicopter on the surface of the water. Companies should provide personal locator beacons for pilots, passengers, and life rafts. Life rafts should be externally mounted on the helicopters. Where appropriate, engine and vibration monitoring equipment should be installed to detect incipient failure.

## Figures and Tables

**FIGURE:**
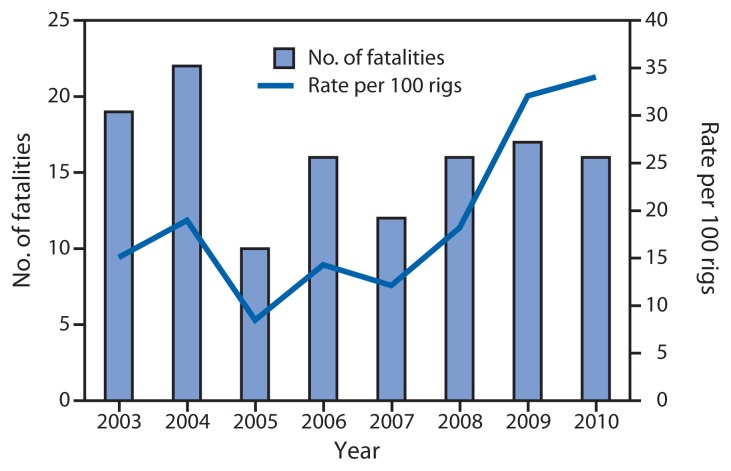
Number and rate of fatal injuries among workers involved in offshore oil and gas operations (N = 128), by year — United States, 2003–2010^*^ **Sources:** U.S. Department of Labor, Bureau of Labor Statistics, Census of Fatal Occupational Injuries. Baker Hughes, Inc., North America Rotary Rig Count. ^*^ Significant increase in fatality rate during 2003–2010 (linear regression χ^2^ = 20.66; p<0.01). Fatality rate calculated per 100 active drilling rigs, which include fixed semisubmersible drilling rigs, mobile offshore drilling units, and drillships, but exclude producing platforms.

**TABLE:** Number and percentage of fatal injuries among workers involved in offshore oil and gas operations, by event — United States, 2003–2010

Event	No.	(%)
Transportation events	65	(50.8)
Aircraft events*	49	(38.3)
Water vehicle events	16	(12.5)
Contact with objects and equipment	21	(16.4)
Fires and explosions	17	(13.3)
Exposure to harmful substances/environments	16	(12.5)
Other event types	9	(7.0)
**Total**	**128**	**(100.0)**

**Source:** U.S. Department of Labor, Bureau of Labor Statistics, Census of Fatal Occupational Injuries.

* All involved helicopters.
